# Knowledge, Misconceptions and Motivations Towards Blood Donation Among University Students in KSA

**DOI:** 10.12669/pjms.296.4137

**Published:** 2013

**Authors:** Mukhtiar Baig, Hamed Habib, Abdullah H. Haji, Faisal T. Alsharief, Abdulelah M. Noor, Riyadh G. Makki

**Affiliations:** 1 Mukhtiar Baig, PhD, Professor in Clinical Biochemistry, Faculty of Medicine Rabigh, King Abdulaziz University, Jeddah, Kingdom of Saudi Arabia.; 2 Hamed Habib, FRCP, Dean Faculty of Medicine Rabigh, Associate Professor, Department of Pediatrics, King Abdulaziz University, Jeddah, Kingdom of Saudi Arabia.; 3 Abdullah H. Haji, Fourth year Students, King Abdulaziz University, Jeddah, Kingdom of Saudi Arabia.; 4 Faisal T. Alsharief, Fourth year Students, King Abdulaziz University, Jeddah, Kingdom of Saudi Arabia.; 5 Abdulelah M. Noor, Fourth year Students, King Abdulaziz University, Jeddah, Kingdom of Saudi Arabia.; 6 Riyadh G. Makki, Fourth year Students, King Abdulaziz University, Jeddah, Kingdom of Saudi Arabia.

**Keywords:** Blood Donation, Knowledge, Misconceptions, Motivations

## Abstract

***Objective:*** To determine the knowledge, misconceptions and motivations towards blood donation among university students in KSA.

***Methods:*** This cross sectional study was carried out at the King Abdulaziz University, Rabigh campus, Jeddah, KSA. A total of 326 adult males were interviewed and each individual completed a questionnaire in Arabic language on various aspects of blood donation. Data was analyzed using SPSS-16.

***Results:*** Out of 326 individuals, 264 (80.98%) were non donors and 62 (19.02%) were donors, 13% donated once, 5% donated twice and 1% donating regularly. Regarding the knowledge part of the questionnaire many of the respondents did not have the basic knowledge and the two common sources of information for blood donation were friends (53%), and TV (24%). The major motivations for donors were to help family or friend (30%), saving others lives (28%), religious reasons (20%) and altruism (12%). Among the respondents the most prevalent misconception was contracting infection like HIV or Hepatitis B&C (26%).

***Conclusion:*** The knowledge of blood donation is not up to the mark and many misconceptions exist among young Saudi University students.

## INTRODUCTION

Blood donation is very crucial in saving lives. In the Kingdom of Saudi Arabia (KSA) health service has been widened and has undergone very swift transformation. The state-of-the art hospitals are providing free and highly specialized services, like open heart surgery, transplant surgeries, cancer treatment, and also providing blood to patients of bleeding disorders, and other hematological disorders.^1^ All these services require ample and continuous blood supply from donors. Another imperative factor in KSA is road traffic accidents which needs lot of blood on emergency basis. The major challenge the blood transfusion services are facing is to meet the increasing demand of the blood and its components constant supply. The blood donation is the only source of blood but the recruitment of voluntary, non remunerated donors is the most important challenge throughout the world.^2^ According to the World Health Organization (WHO) data blood donation rate is high in high-income countries and low in middle and low-income countries 39.2, 12.6, 4.0 donations per 1000 population respectively.^3^

Many previous reports have shown that people have insufficient knowledge, diverse attitude and many misconceptions about the blood donation.^4^^-^^9^ The donation of blood from young students is preferred because the risk of acquiring the blood transmitted diseases from blood donated by the students is less than other groups.^10^ The survey regarding knowledge, attitude and misconceptions may help to blood donation centers to develop their future policies to motivate people to donate blood and to urge donors to keep on donating blood on a regular basis and to inspire non-donors to start donating blood.^1^ Therefore this study was undertaken to determine the knowledge, reasons for not donating blood, misconceptions and motivations towards blood donation among a sample of university students in KSA.

## METHODS

This cross sectional study was carried out at the King Abdulaziz University, Rabigh campus, KSA, during February and March 2013. 

A self-administered questionnaire was prepared in English language with the help of previous similar studies. The questionnaire was translated into Arabic language and pretested and verified for error on group of fifty students. A total of 326 adult males were interviewed and each individual completed a questionnaire containing epidemiological data and questions regarding knowledge, misconceptions and motivations concerning blood donation. The age of the respondents ranged between 18 and 28 years (mean age 22 ± 2.3 years). All respondents were informed about the survey and data was analyzed using SPSS-16. 

## RESULTS

Out of 326 individuals, 264 (80.98%) were non donors and 62 (19.02%) were donors, 13% donated once, 5% twice and 1% donating regularly. Regarding the awareness part of the questionnaire many of the respondents did not have basic knowledge and two more common sources of information for blood donation were friends (53%), and TV (24%) as shown in Table-I. 

Regarding the knowledge and attitude part of the questionnaire many of the respondents had lack of knowledge (Table-II). Many of the responded replied that a token gift/money should be given to donors (30%). The majority of the respondents intended to donate blood if blood donation camps are arranged in the university premises (70%) and if a family member, relative, or friend needs (53%) (Table-II).

**Table-I:** Awareness regarding blood donation

*Questions *	*No of respondents (%)*
*Amount of blood drawn for each donation*	
<500ml	69(21.2)
500-1000 ml	60(18.4)
Don’t know	197(60.4)
*Age limit for blood donation*	
17 -35 yr	142(43.6)
17-45 yr	159(48.8)
17-60 yr	25(7.7)
*Minimum weight of donors*	
50 kg	95(29.1)
51-60 kg	89(27.3)
61-70kg	99(30.4)
>70 kg	43(13.2)
*Interval between two successive donations*	
3 months	116(35.6)
6 months	132(40.5)
9 months	28(8.6)
12 months	50(15.3)
*Duration of donated blood volume replacement *	
< One day	64(19.6)
One week	66(20.2)
One month	71(21.8)
Never	125(38.3)
*Source from where you heard about blood donation*	
Friends & relatives	174(53.4)
Blood bank staff	30(9.2)
Newspaper & book	23(7.1)
Television	77(23.6)
None	22(6.7)

**Table-II:** Knowledge and attitude regarding blood donation

*Knowledge and attitude towards blood donation*	*Yes* *%*	*No* *%*
Blood is screened for AIDS, Hepatitis B & C before transfusion	49	51
All surgical procedures require blood transfusion	39	61
Blood can be used in cancer treatment	55	45
Blood is required in emergencies	56	44
Blood can be stored	92	8
Blood can be donated while keeping a fast	29	71
A token gift/money should be given to donors	30	70
Blood should be imported from abroad	6	94
I will donate blood if a family, relative, or friend needs	53	47
I would donate blood if blood donation camp arrange in the university premises	70	30

**Table-III:** Reasons for not donating blood, and motivations & misconceptions regarding blood donation.

*Reasons for not donating blood, motivations & misconceptions*	*%*
*Reasons for not donating blood*	
Unknown Fear	7
Unaware of collection facility	4
Don’t have enough time to donate	3
Concerned about sterility of equipments	11
No one ever asked for donation	45
Never thought to donate	3
Don’t have enough information	4
Believe that there is no need for blood	2
Apprehension about feeling weakness after donation	6
They would take too much blood	3
Afraid of the sight of blood	2
Afraid of the needle prick	5
Not eligible on medical ground	1
Process is long and boring	2
No specific reason	2
*Motivation for donors *	
To help family or friend in need	30
Altruism	12
Personally asked	5
Money/gift	1
To learn about AIDS/Hepatitis B&C status	4
Religious reasons	20
Save life	28
*Misconceptions *	
Donor has risk for contracting infection like HIV or Hepatitis B& C	26
Lead to infertility and loss of vitality	7
Lead to permanent weakness/ anemia	11
Lead to fainting or death	10
Affect physical strength	24
A painful procedure	16
Harmful to health	6

The most prevalent misconception among the respondents was that the blood donor has risk for contracting infection like HIV or Hepatitis B&C infection (26%), followed by others shown in Table-III. Among the non-donor respondents, the most common reasons for not donating blood was not approached by anybody' for blood donation (45%) followed by concerned about sterilization of equipments (11%), unknown fear (7%), and many others shown in Table-III. Questions regarding the motivation for donors were answered as, to help family or friend (30%), it saves life (28%), religious reasons (20%) and others shown in Table-III.

## DISCUSSION

The current study found that the knowledge of the respondents was not up to the mark (Table I & II). These results are similar to several previous studies.^4^^-^^6^^,^^8^^,^^9^ In contrast to our finding several studies have shown higher level of knowledge and more positive attitude regarding blood donation among University students.^11^^-^^13^ A recent survey among Jordanian University students found poor knowledge of the students regarding blood donation.^14^ Giri & Phalke, (2012)^11^ found overall good knowledge in the respondents, but that survey was carried out in the institute of Medical Sciences that could be one of the reasons of students better knowledge. Safizadeh et al., (2007)^15^ in Iran found that awareness was not good regarding blood donation while a study in Pakistan reported that respondents don’t have good knowledge and awareness.^7^ It is a very surprising fact that in this advanced era when modern gadgets and communication tools are in the access of every person but the several studies have documented lack of knowledge among people regarding blood donation. This is a point to ponder for the policy makers and blood transfusion services because it is adversely affecting the blood donation rate.

In present study friends and television were found to be the more common sources of information for blood donation (53%) and (24%) respectively, while Al-Dress, (2008)^5^ stated that the source of awareness for majority of the participants about blood donation was from daily news papers and/or TV and internet. Likewise, another study in KSA described that the blood bank staff and friends were the major source of information.^4^ A study in India found that the majority of the participants acquainted information about blood donation from television (45.2%), newspapers (39.8%), and radio (9.2%).^16^ A study in Jordan revealed that participants obtained information about blood donation from medical staff (27%), friends (25%) Television/radio (16.4%), and newspapers/books (22%).^8^ The television and radio were found to be the major source of information in Pakistan and Iran.^7^^,^^10^

The promotion of such awareness has been found a main motivating factor among United States blood donors.^17^ One important finding of the study is that the 70% of the respondent want to donate blood if blood donation camps are arranged in the University premises. So blood transfusion services should organize blood donation camps in the Universities on regular basis and it should be mandatory from Health Department/Higher Education Department that every Medical College and University must have one blood donor society and that must arrange at least one Blood Donor Camp in the University premises in a year. It seems that by this way the chances of increasing blood donor pool will be augmented.

In present study 80% of the respondents had never donated blood and these results are in accordance to other studies.^5^^-^^6^^,^^9^ Our study observed that the most common reason for not donating blood was that nobody approached them. The other reasons given for refraining from blood donation were concerned about sterilization of equipments, unknown fear, feeling weakness after donation, afraid of the needle prick etc. Our results are similar to several other studies.^1^^,^^6^^,^^9^ A study in a poor locality in India observed the most common reason of not donating blood was the thinking that blood donation has harmful effects (50%) and many people said that they don’t have any reason to donate blood (25%).^18^ There is utmost need to approach those people who have never been approached and they intend to donate blood.

The present study shows that the major motivators for donors were to help family or friend, for saving others lives, religious reasons and altruism. Several studies have observed altruism as an important motivating factor among donors.^7^^,^^18^^,^^19^ A previous study in Saudia observed that 91% of the donors donate blood because of the religious reason.^1^ In a recent study in India the majority of donors (57%) agreed to donate blood to help family or friend, others motivating factors were altruism (16%), to know human immunodeficiency virus (HIV) status (8%) and to take some incentive (6%)^17^ but a study in Africa, revealed that personal gain as the most important motivational factor (93%).^20^

The current study observed that 30% of the respondents were in favor of incentive like money or token gift. Salaudeen & Odeh (2011)^6^ observed that 29% preferred some incentives like wrist bands, pens, T-shirts, and diaries. In another study 41% of the participants preferred a certificate as an incentive, 13.6% preferred money. In a study in KSA majority of donors (85%) disapproved the idea of giving money to donors but 63% accepted the idea of a token gift like watch, pen, Arabian head dress (Ghutra).^1^ A study in Iran found that 25.3% respondents said that incentives should be given to encourage them to donate.^21^ Glynn et al., (2003)^22^ in America demonstrated that health incentives like blood investigations (serum cholesterol, prostatic specific antigen and complete blood count), souvenirs and/or lottery tickets increased the blood donors.

The current study revealed that among the respondents the most prevalent misconception was that the blood donor has risk for contracting infection like HIV or Hepatitis B&C followed by others (Table-III). Our results are similar to several studies.^6^^,^^13^^,^^16^^,^^20^^,^^23^ A study in Pakistan reported that 10.2% of the participants avoid to donate blood because of the fear of transmission of diseases.^7^ Similarly, a study reported misconception of acquiring AIDS and hepatitis due to blood donation among the French population.^24^ A study in Nigeria observed that 52.4% students have misconception of acquiring AIDS and hepatitis by blood donation.^20^ An Iranian study mentioned that majority of the students (66.6%) believed that blood donation is a way for transmitting infections.^15^ We suggest that for the success of blood donation campaigns it is necessary to remove people misconceptions and this could be achieved by better planning.

## CONCLUSION

The knowledge of blood donation is not up to the mark and many misconceptions are prevailing among young Saudi University students.

## Notes

***Source of funding: ***None.

## RECOMMENDATIONS

We recommend proper and regular motivational campaigns in Colleges and Universities to recruit more voluntary and non-remunerated donors. It is also recommended that to enhance awareness and remove misconceptions, a detailed chapter about blood donation and transfusion should be incorporated in Science subject at school level so that students have all necessary information.
